# ADCK1 Regulates Mitochondrial Bioenergetics in Hepatocellular Carcinoma In Vitro

**DOI:** 10.3390/ijms27187984

**Published:** 2026-09-08

**Authors:** Noel Jacquet, Yunfeng Zhao

**Affiliations:** Department of Pharmacology, Toxicology & Neuroscience, LSU Health Sciences Center, 1501 Kings Highway, Shreveport, LA 71130, USA

**Keywords:** AarF domain-containing kinase 1, ADCK1, liver cancer, hepatocellular carcinoma, mitochondria, cellular bioenergetics, cellular metabolism

## Abstract

Hepatocellular carcinoma (HCC) is characterized by profound metabolic reprogramming and mitochondrial dysfunction, yet the molecular regulators underlying these alterations remain incompletely understood. AarF domain-containing kinase 1 (ADCK1) is an evolutionarily conserved protein associated with mitochondrial function, but its role in HCC bioenergetics has not been defined. In this study, we investigated the effects of ADCK1 on mitochondrial metabolism using CRISPR/Cas9-mediated ADCK1 knockout in HepG2 and SNU-449 HCC cells. Mitochondrial respiration, glycolytic activity, ATP production, lactate generation, mitochondrial membrane potential, and superoxide production were assessed following ADCK1 KO. ADCK1 KO resulted in marked reductions in basal and maximal mitochondrial respiration, ATP-linked respiration, glycolytic activity, intracellular ATP, and lactate production in both HCC cell models. ADCK1 KO also reduced mitochondrial membrane potential in a clone-dependent manner. Despite these profound bioenergetic defects, mitochondrial superoxide production was not consistently altered across the knockout clones. These findings indicate that ADCK1 supports both oxidative phosphorylation and glycolytic metabolism and is required for maintenance of bioenergetic homeostasis in HCC cells. Collectively, our results identify ADCK1 as a previously unrecognized regulator of HCC mitochondrial metabolism.

## 1. Introduction

Hepatocellular carcinoma (HCC) is the most common primary liver malignancy and remains one of the leading causes of cancer-related mortality worldwide. Despite advances in surveillance and therapeutic approaches, the prognosis for patients with advanced HCC remains poor because of its aggressive nature, high rates of recurrence, and limited treatment options. HCC development is driven by a complex interplay of genetic, metabolic, and environmental factors that collectively promote chronic liver injury, inflammation, and malignant transformation. HCC is characterized by profound mitochondrial dysfunction, which contributes to tumor progression and altered bioenergetics [1,2,3,4,5,6].

Mitochondrial dysfunction has emerged as a hallmark of HCC progression. Cancer cells undergo profound metabolic reprogramming to support rapid proliferation, resulting in altered mitochondrial bioenergetics, increased oxidative stress, and disrupted energy metabolism [7,8,9]. Major risk factors for HCC, including chronic hepatitis B virus (HBV) and hepatitis C virus (HCV) infection, nonalcoholic fatty liver disease (NAFLD), nonalcoholic steatohepatitis (NASH), and cirrhosis, contribute to a unique tumor microenvironment characterized by chronic inflammation, hypoxia, and elevated reactive oxygen species (ROS). These pathological conditions promote mitochondrial dysfunction and further accelerate tumor progression [10,11,12,13,14]. Although mitochondrial dysfunction is recognized as a critical component of HCC pathogenesis, the molecular mechanisms regulating mitochondrial homeostasis in HCC remain incompletely understood [2,11,15,16].

The AarF domain-containing kinase (ADCK) family comprises a family of evolutionarily conserved, putative mitochondrial proteins that are implicated in regulating mitochondrial function and cellular bioenergetics. Several members of this family have been associated with the biosynthesis of coenzyme Q (CoQ), an essential component of the mitochondrial electron transport chain that facilitates oxidative phosphorylation and functions as a major cellular antioxidant [17,18]. Consequently, defects in ADCK proteins have been linked to impaired mitochondrial respiration [17], altered ATP production [18], and increased oxidative stress [19]. While multiple ADCK family members have established roles in CoQ metabolism and mitochondrial homeostasis, the biological function of ADCK1 remains largely unknown [20,21,22,23,24].

ADCK1 is highly conserved across species, including yeast, *Drosophila melanogaster*, *Caenorhabditis elegans*, and mammals, suggesting that it performs an evolutionarily important cellular function. Studies in *Drosophila* have demonstrated that ADCK1 is both necessary and sufficient for normal larval development and viability. Furthermore, ADCK1 KO results in severe mitochondrial dysfunction and premature lethality, highlighting its importance in maintaining mitochondrial integrity. However, whether ADCK1 performs similar functions in mammalian cells, particularly in HCC, remains unclear [25,26].

Previous work from our laboratory generated stable ADCK1 knockout (KO) HCC cell lines in both HepG2 and SNU-449 cells. In contrast to the lethality observed in *Drosophila*, complete ADCK1 KO was compatible with the survival of these human HCC cell lines, indicating that ADCK1 is not essential for viability under standard cell culture conditions. These models provide a unique opportunity to investigate ADCK1’s role in regulating mitochondrial function in human liver cancer.

In the present study, we utilized previously established HepG2 and SNU-449 ADCK1 KO cell lines to determine how ADCK1 influences mitochondrial bioenergetics in HCC. By characterizing the effects of ADCK1 KO on mitochondrial function, this work aims to define previously unrecognized roles for ADCK1 in cancer cell metabolism and to evaluate its potential as a regulator of mitochondrial homeostasis in HCC. Improved understanding of ADCK1 biology may provide new insights into the metabolic mechanisms underlying HCC progression and identify potential therapeutic opportunities targeting mitochondrial dysfunction.

## 2. Results

### 2.1. ADCK1 Inhibition Impairs Mitochondrial Respiration in Hepatocellular Carcinoma Cells

To determine whether ADCK1 contributes to mitochondrial bioenergetics in hepatocellular carcinoma (HCC), mitochondrial respiration rates in HepG2 and SNU-449 cells were measured using the Seahorse XF24 Extracellular Flux Analyzer. We detected the oxygen consumption rate (OCR) throughout a mitochondrial stress test following sequential administration of oligomycin, FCCP, and rotenone/antimycin A to evaluate multiple aspects of oxidative phosphorylation.

**Figure 1:**
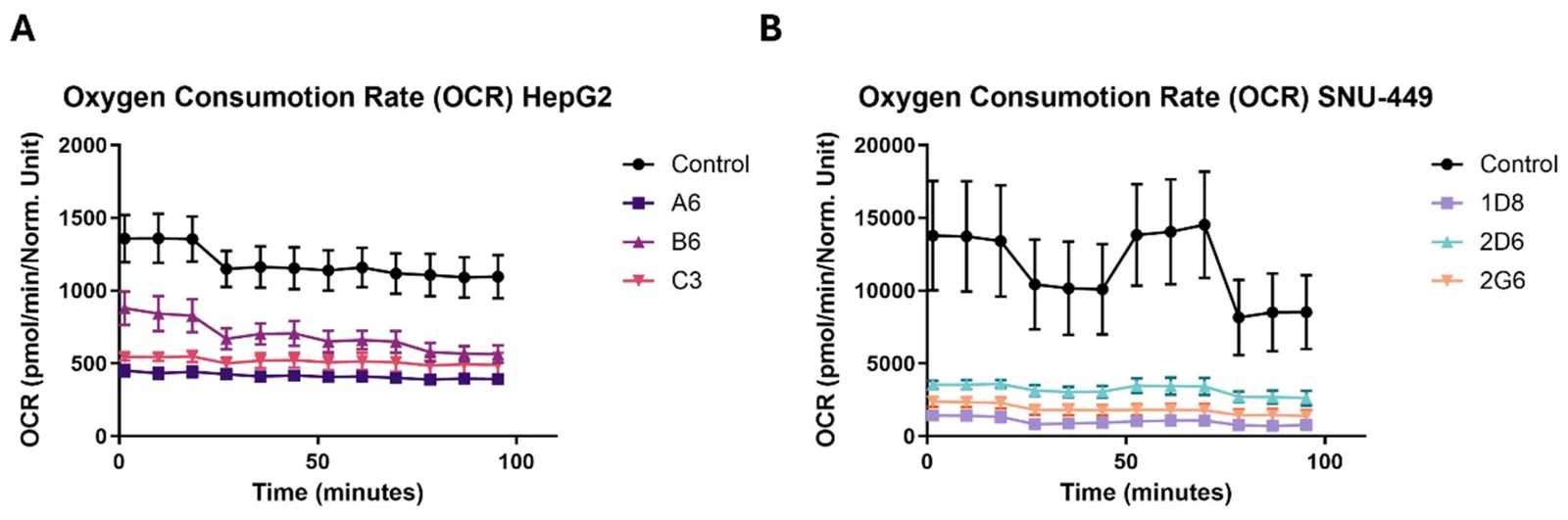
ADCK1 Knockout Reduces Total Mitochondrial Respiration in HCC Cells. Oxygen consumption rates (OCR) were measured in HepG2 (**A**) and SNU-449 (**B**) cells using a Seahorse XF24 Extracellular Flux Analyzer. A mitochondrial stress test was conducted by sequential administrating oligomycin, FCCP, and rotenone/antimycin A to assess mitochondrial respiratory function. ADCK1 knockout (KO) significantly reduced OCR throughout the assay in both HepG2 and SNU-449 cells compared with control cells.

The OCR data demonstrated a pronounced reduction in mitochondrial respiration following ADCK1 KO in both HCC cell lines (Figure 1A,B; N = 5, *p* < 0.0001). Compared with vector control cells, ADCK1 knockout clones displayed lower oxygen consumption under basal conditions and maintained significantly reduced respiration following each pharmacological treatment of the electron transport chain.

**Figure 2:**
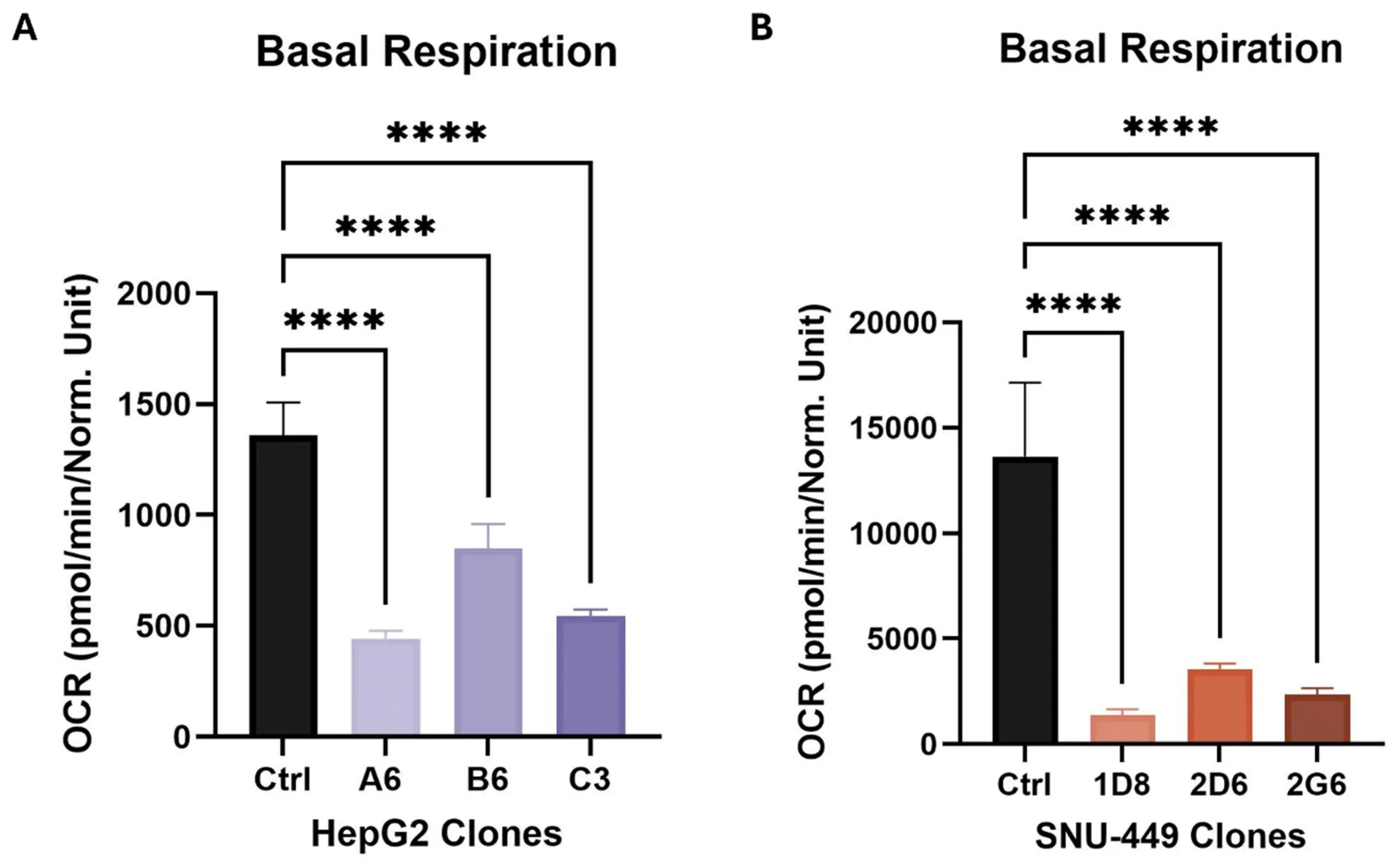
ADCK1 Knockout Impairs Basal Mitochondrial Respiration. Basal respiration was quantified from Seahorse XF24 OCR measurements obtained prior to mitochondrial stressor administration in HepG2 (**A**) and SNU-449 (**B**) cells. ADCK1 KO significantly reduced basal respiration in all KO clones compared with controls (both **** *p* < 0.0001).

**Figure 3:**
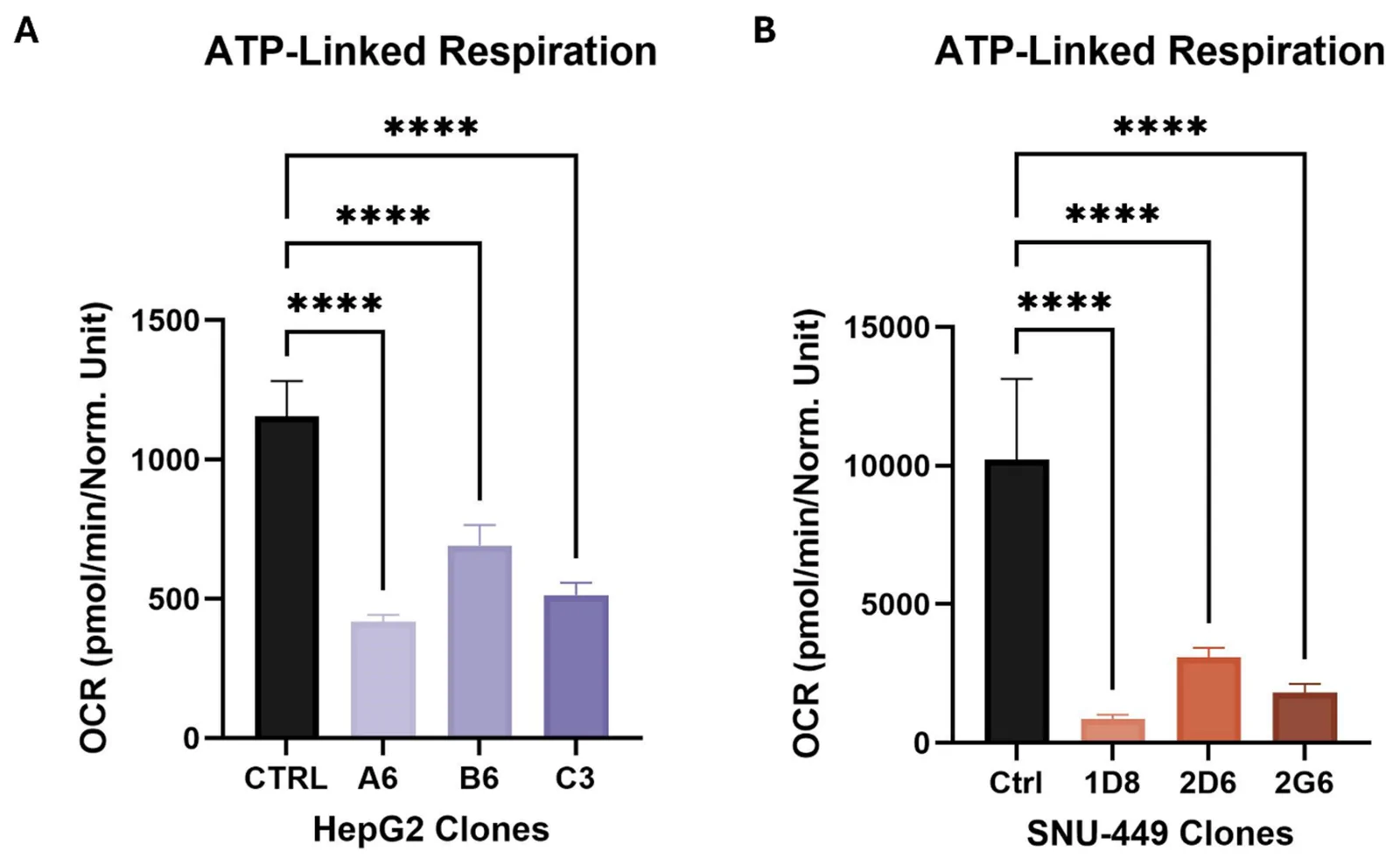
ADCK1 Knockout Decreases ATP-Linked Respiration. ATP-linked respiration was determined from Seahorse XF24 OCR measurements following oligomycin treatment, which inhibits ATP synthase (Complex V). ADCK1 KO significantly reduced ATP-linked respiration in HepG2 (**A**) and SNU-449 (**B**) cells compared with controls (both **** *p* < 0.0001).

Basal respiration, which reflects the energetic demands of cells under resting conditions, was significantly decreased in all ADCK1 knockout clones compared with controls (Figure 2A,B; N = 5; both **** *p* < 0.0001), suggesting diminished mitochondrial reserve capacity. After inhibiting ATP synthase with oligomycin, the ATP-linked respiration rate was calculated to determine the proportion of oxygen consumption coupled to ATP production. ADCK1 knockout significantly reduced ATP-linked respiration in both HepG2 and SNU-449 cells (Figure 3A,B; N = 5; both **** *p* < 0.0001), indicating that oxidative ATP synthesis is markedly impaired in the absence of ADCK1 expression.

**Figure 4:**
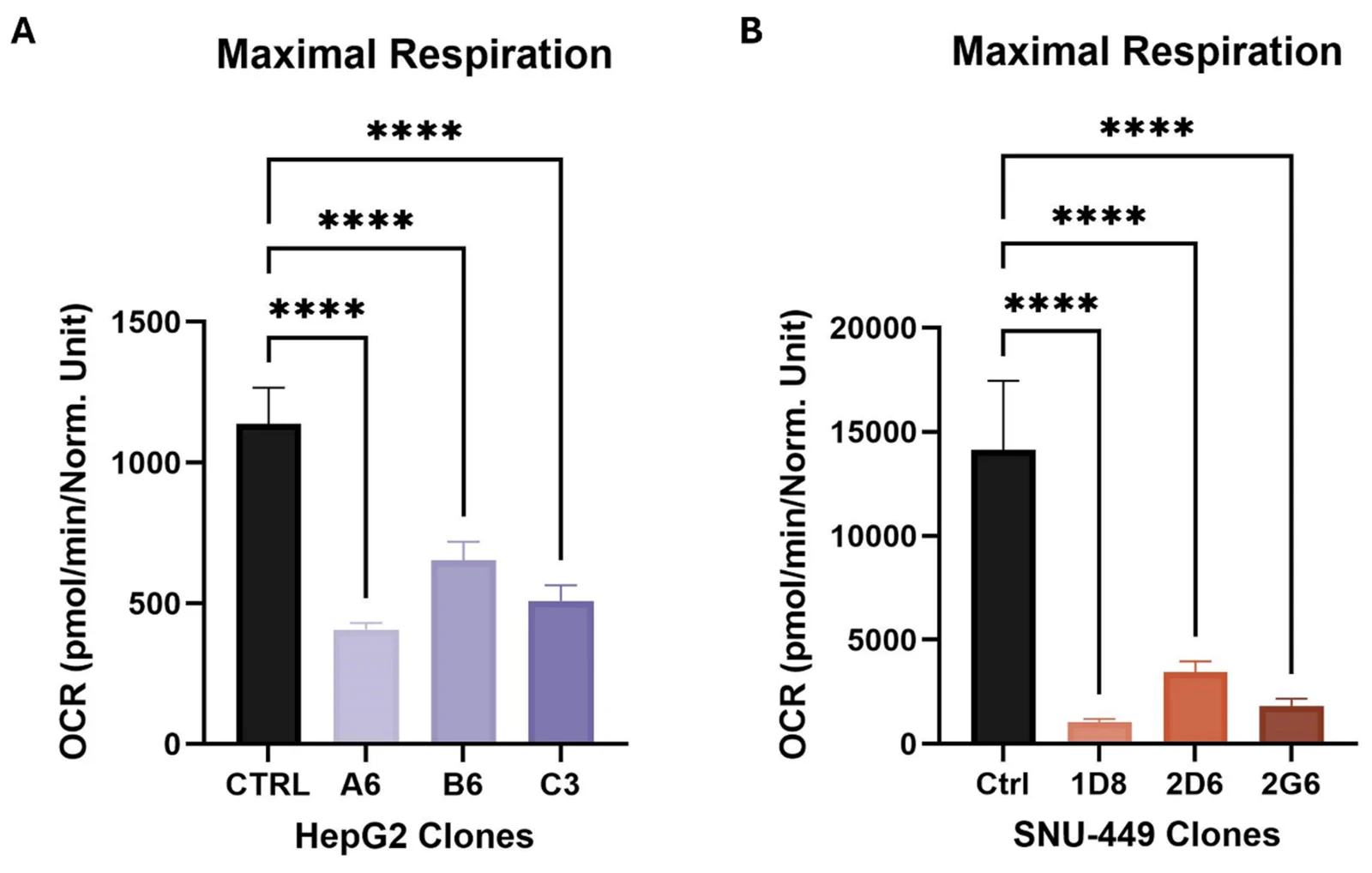
ADCK1 Knockout Reduces Maximal Mitochondrial Respiratory Capacity. Maximal respiration was determined by Seahorse XF24 analysis following FCCP treatment, which uncouples oxidative phosphorylation and induces maximal electron transport chain activity. ADCK1 KO significantly decreased maximal respiration in HepG2 (**A**) and SNU-449 (**B**) cells compared with controls (both **** *p* < 0.0001).

To evaluate maximal respiratory capacity, cells were treated with FCCP to uncouple oxidative phosphorylation and stimulate maximal electron transport chain activity. ADCK1 knockout cells exhibited significantly lower maximal respiration than vector controls in both HepG2 and SNU-449 cell lines (Figure 4A,B; N = 5; both *p* < 0.0001). The reduced maximal OCR indicates that ADCK1-KO cells possess a diminished respiratory reserve, suggesting an impaired ability to respond to increased energetic demand or metabolic stress. Collectively, these findings demonstrate that ADCK1 plays an essential role in maintaining mitochondrial oxidative phosphorylation in HCC cells. ADCK1 KO compromises basal respiration, ATP-linked respiration, and maximal respiratory capacity, indicating a broad disruption of mitochondrial respiratory function.

### 2.2. ADCK1 Knockout Suppresses Glycolytic Activity

**Figure 5:**
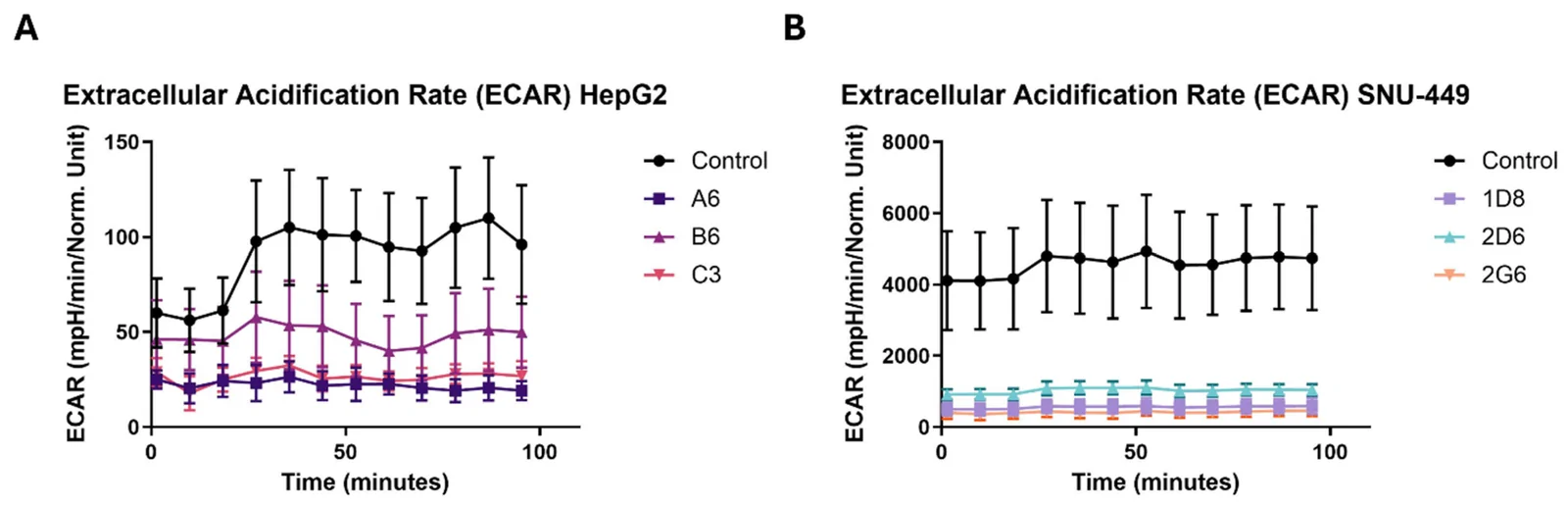
ADCK1 Knockout Reduces Extracellular Acidification in HCC Cells. The extracellular acidification rates (ECAR) were measured in HepG2 (**A**) and SNU-449 (**B**) cells using a Seahorse XF24 Extracellular Flux Analyzer during the mitochondrial stress test. Oligomycin was used to evaluate glycolytic responses. ADCK1 KO significantly reduced ECAR throughout the assay in both cell lines compared with controls.

**Figure 6:**
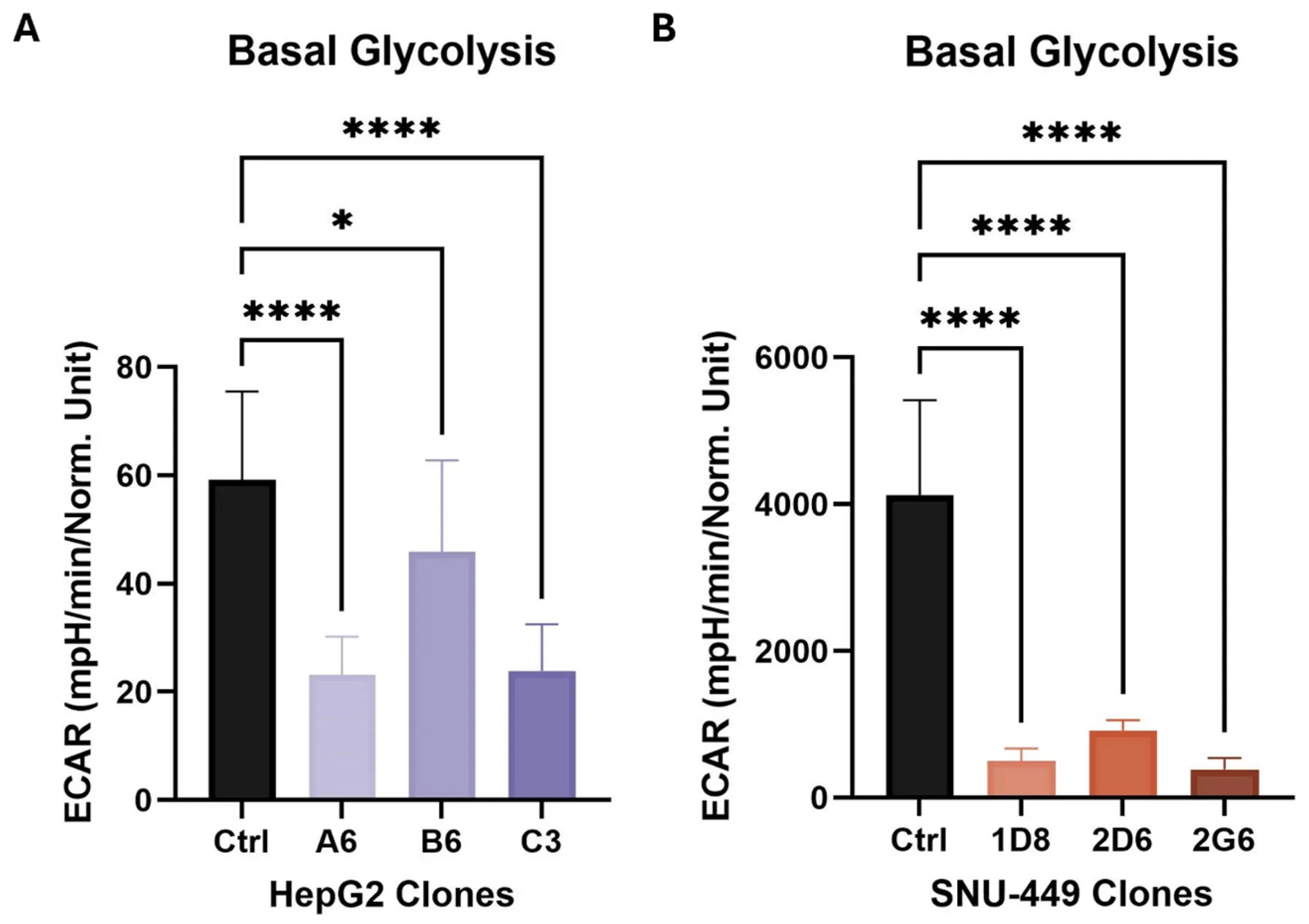
ADCK1 Knockout Impairs Basal Glycolytic Activity. Basal glycolysis was determined from ECAR measurements obtained before mitochondrial stressor administration in HepG2 (**A**) and SNU-449 (**B**) cells. ADCK1 KO significantly reduced basal glycolytic activity in all KO clones compared with controls (**** *p* < 0.0001;* *p* < 0.05).

Because mitochondrial dysfunction is accompanied by compensatory changes in glycolysis, extracellular acidification rate (ECAR) was measured simultaneously during the mitochondrial stress assay. Surprisingly, ADCK1 KO did not induce glycolytic compensation. Instead, ECAR was significantly reduced throughout the assay in both HepG2 and SNU-449 knockout cells compared with controls (Figure 5A,B; N = 5; both **** *p* < 0.0001). Basal glycolysis, measured prior to the addition of mitochondrial inhibitors, was significantly decreased in every ADCK1 knockout clone examined (Figure 6A,B; N = 5; **** *p* < 0.0001; * *p* < 0.05). This reduction indicates that glycolytic activity is impaired even under resting conditions.

**Figure 7:**
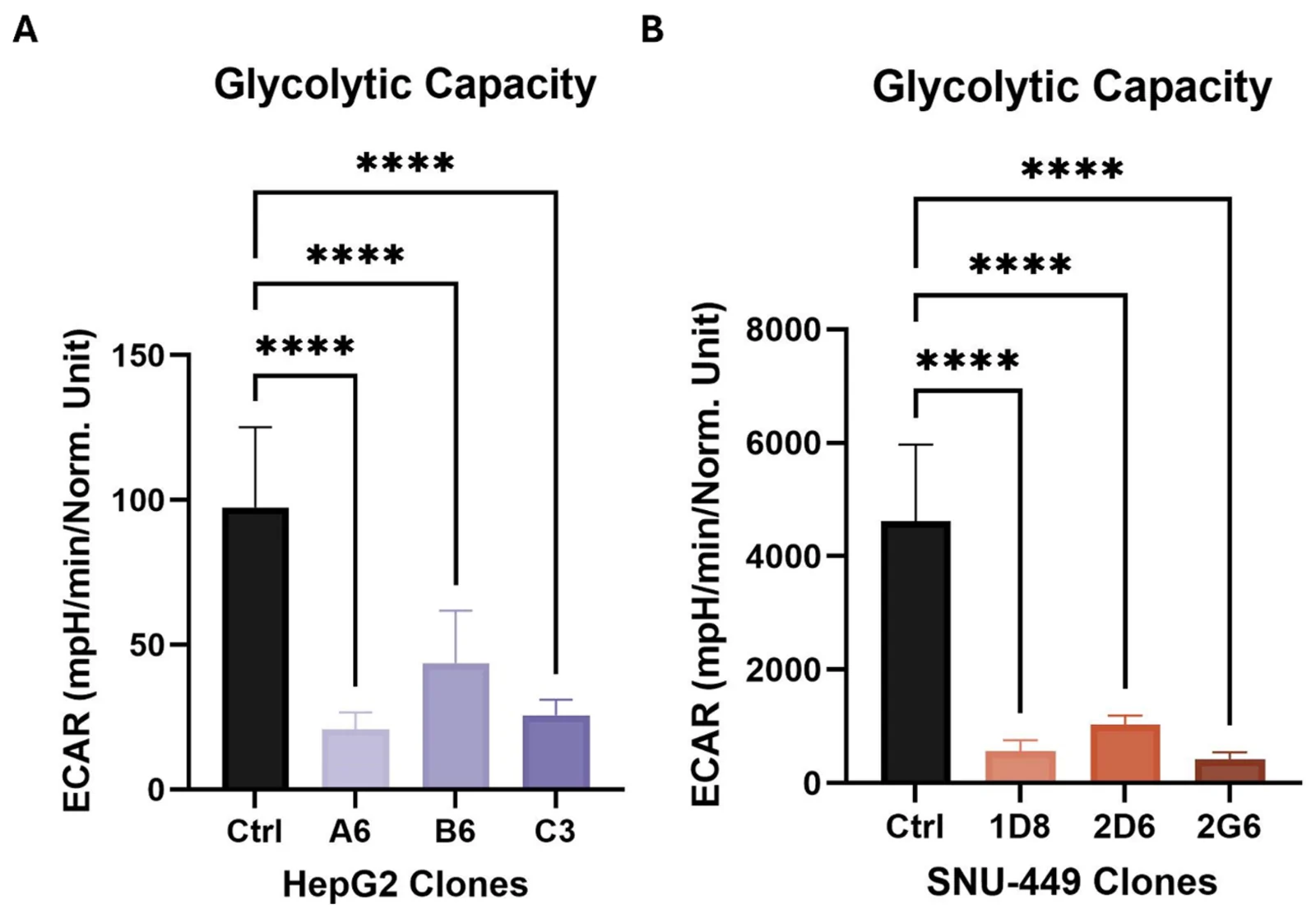
ADCK1 Knockout Decreases Glycolytic Capacity. Glycolytic capacity was measured by Seahorse XF24 analysis after the oligomycin treatment, which inhibits mitochondrial ATP production and promotes maximal glycolytic flux. ADCK1 KO significantly reduced glycolytic capacity in HepG2 (**A**) and SNU-449 (**B**) cells compared with controls (both **** *p* < 0.0001).

After the oligomycin treatment, cells normally increase glycolytic flux to compensate for the loss of mitochondrial ATP production. In contrast, ADCK1 knockout cells displayed a significantly reduced glycolytic capacity in both HepG2 and SNU-449 cell lines (Figure 7A,B; N = 5; both *p* < 0.0001). The inability of knockout cells to increase glycolysis following mitochondrial inhibition suggests reduced metabolic flexibility and an impaired capacity to adapt to energetic stress.

Taken together, the OCR and ECAR analyses demonstrate that ADCK1 KO suppresses both oxidative phosphorylation and glycolysis. Rather than exhibiting the metabolic reprogramming often observed following mitochondrial dysfunction, ADCK1 KO HCC cells adopt a globally metabolically quiescent phenotype characterized by reduced mitochondrial respiration and diminished glycolytic capacity.

### 2.3. ADCK1 Knockout Reduces Cellular ATP and Lactate Production

**Figure 8:**
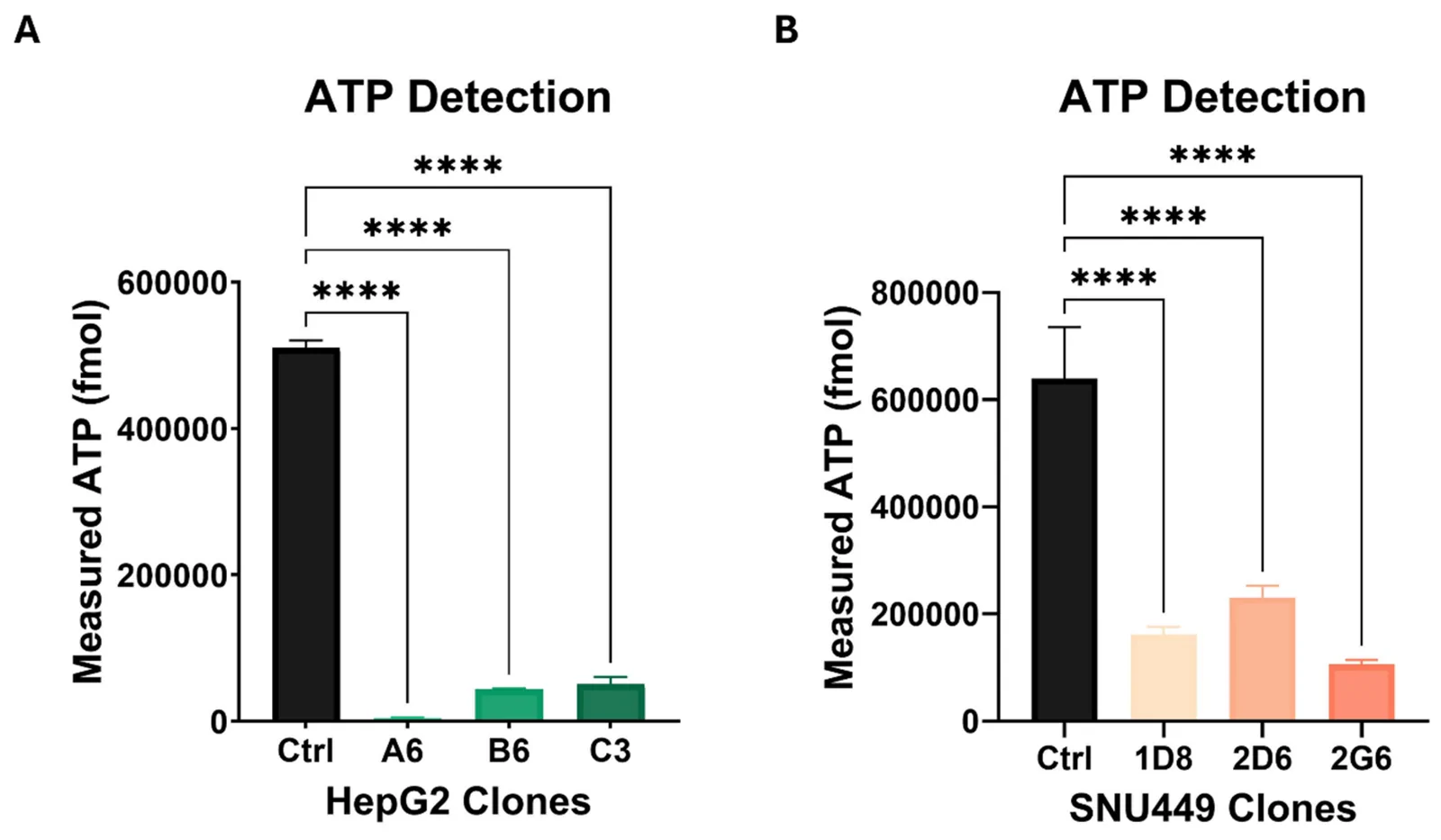
ADCK1 Knockout Decreases Intracellular ATP Production. The intracellular ATP levels were quantified using the Cayman ATP Detection Assay Kit, and luminescence was measured using a BioTek Synergy H1 microplate reader. ADCK1 KO significantly reduced intracellular ATP levels in HepG2 (**A**) and SNU-449 (**B**) cells compared with controls (both **** *p* < 0.0001).

To determine whether the bioenergetic defects observed during Seahorse analysis translated into impaired cellular metabolism, we quantified intracellular ATP and lactate levels in ADCK1 knockout (KO) HCC cells. Intracellular ATP concentrations, measured using a luciferase-based assay (N = 9), were significantly reduced in both HepG2 and SNU-449 ADCK1 KO cells compared with vector controls (Figure 8A,B; both **** *p* < 0.0001). These findings are consistent with the observed reductions in mitochondrial respiration and ATP-linked oxygen consumption, indicating that ADCK1 KO compromises cellular ATP production.

**Figure 9:**
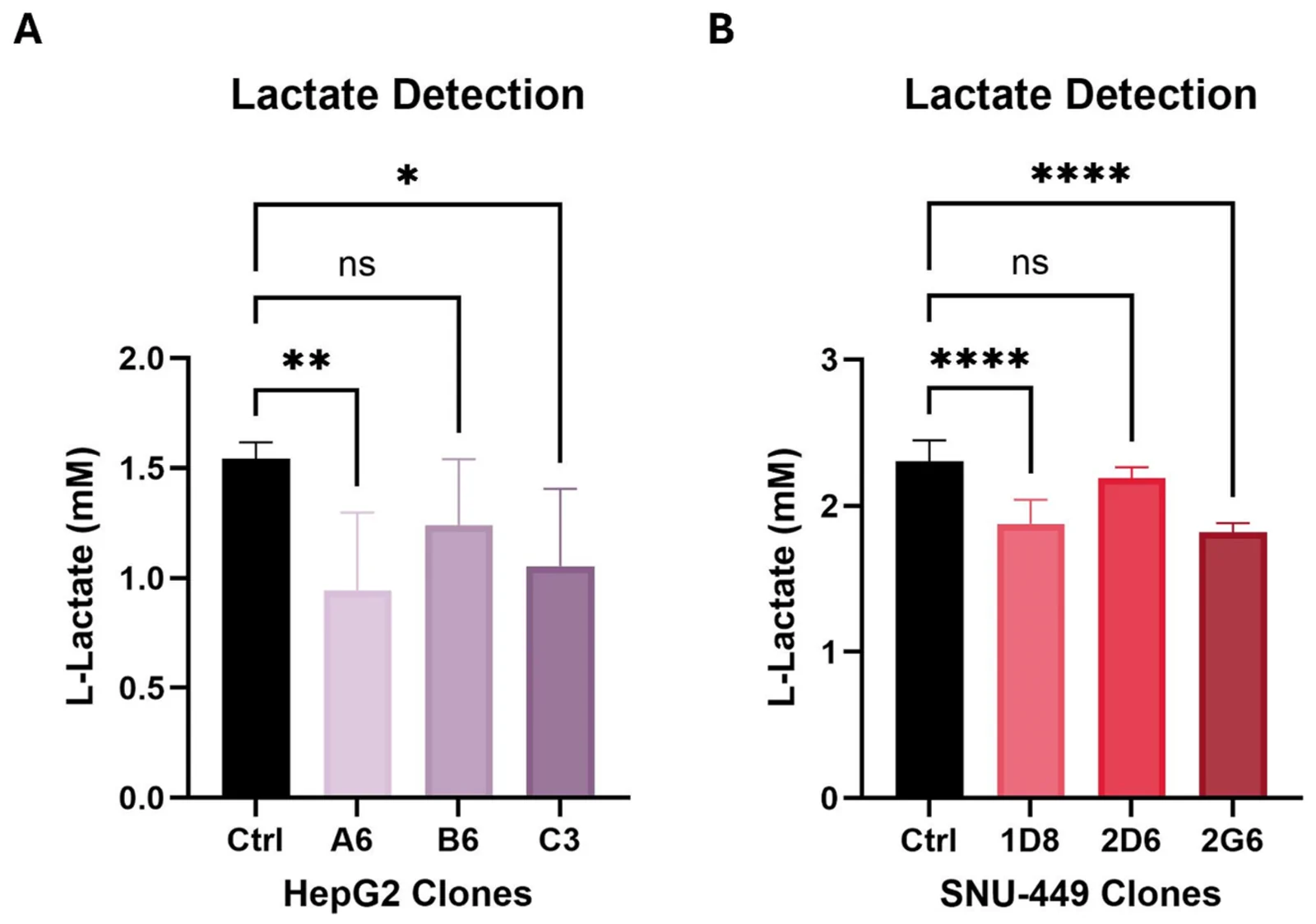
ADCK1 Knockout Reduces Intracellular Lactate Production. The intracellular L-lactate concentrations were measured using the Abcam L-Lactate Colorimetric Assay Kit and quantified with a BioTek Synergy H1 microplate reader. In HepG2 cells (**A**), lactate levels were significantly reduced in the A6 and C3 ADCK1 KO clones (** *p* < 0.001; * *p* < 0.05), whereas the B6 clone exhibited a nonsignificant downward trend. In SNU-449 cells (**B**), the 1D8 and 2G6 ADCK1 KO clones displayed significantly reduced intracellular lactate levels compared with controls (both **** *p* < 0.0001). ns: no significant difference.

To further assess glycolytic metabolism, we measured intracellular lactate production as an independent indicator of glycolytic flux (N = 5–6). Consistent with the reductions in extracellular acidification rate (ECAR) observed during Seahorse analysis, ADCK1 knockout significantly decreased intracellular lactate concentrations in the HepG2 A6 and C3 clones (** *p* < 0.001 and * *p* < 0.05, respectively) and in the SNU-449 1D8 and 2G6 clones (both **** *p* < 0.0001) (Figure 9A,B). Although the magnitude of reduction varied among individual knockout clones, the overall decrease in lactate production across both cell lines supports a suppression of glycolytic activity following ADCK1 KO.

Together, these findings demonstrate that ADCK1 KO disrupts both mitochondrial ATP production and glycolytic output. The reductions in ATP and lactate production complement the Seahorse analyses, providing evidence that ADCK1 KO produces a metabolically quiescent phenotype characterized by impaired oxidative phosphorylation and diminished glycolytic capacity.

### 2.4. ADCK1 Knockout Impairs Mitochondrial Membrane Potential but Produces Modest Changes in Mitochondrial Superoxide

**Figure 10:**
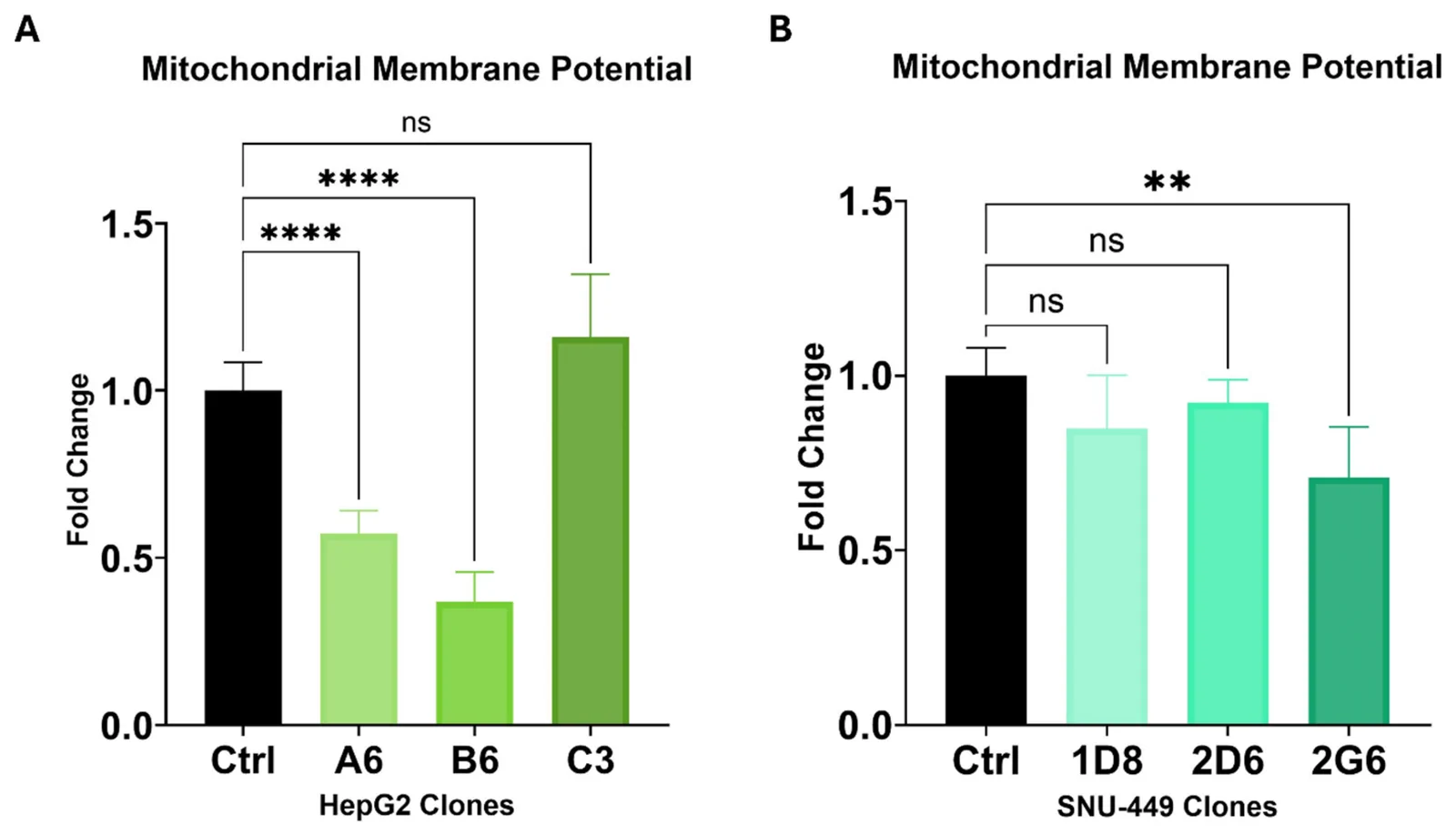
ADCK1 Knockout Disrupts Mitochondrial Membrane Potential. Mitochondrial 1. (**A**) KO significantly reduced TMRE fluorescence in the HepG2 A6 and B6 clones (both **** *p* < 0.0001). (**B**) In SNU-449 cells, a significant reduction in TMRE fluorescence was observed in the 2G6 clone (** *p* < 0.005), consistent with partial depolarization of the mitochondrial membrane in these subpopulations. ns: no significant difference.

To further investigate the effects of ADCK1 KO on mitochondrial function, mitochondrial membrane potential (ΔΨm) and mitochondrial superoxide levels were increased in HepG2 and SNU-449 hepatocellular carcinoma cells. Because maintaining ΔΨm is essential for oxidative phosphorylation and ATP synthesis, TMRE fluorescence intensity was used as an indicator of mitochondrial membrane integrity (N = 6). ADCK1 knockout significantly reduced TMRE fluorescence in the HepG2 A6 and B6 clones (both **** *p* < 0.0001) and in the SNU-449 2G6 clone (** *p* < 0.005) relative to vector control cells (Figure 10A,B). The reduction in TMRE fluorescence indicates partial depolarization of the mitochondrial membrane potential in ADCK1 KO samples, suggesting that ADCK1 contributes to the maintenance of mitochondrial membrane integrity in HCC cells.

**Figure 11:**
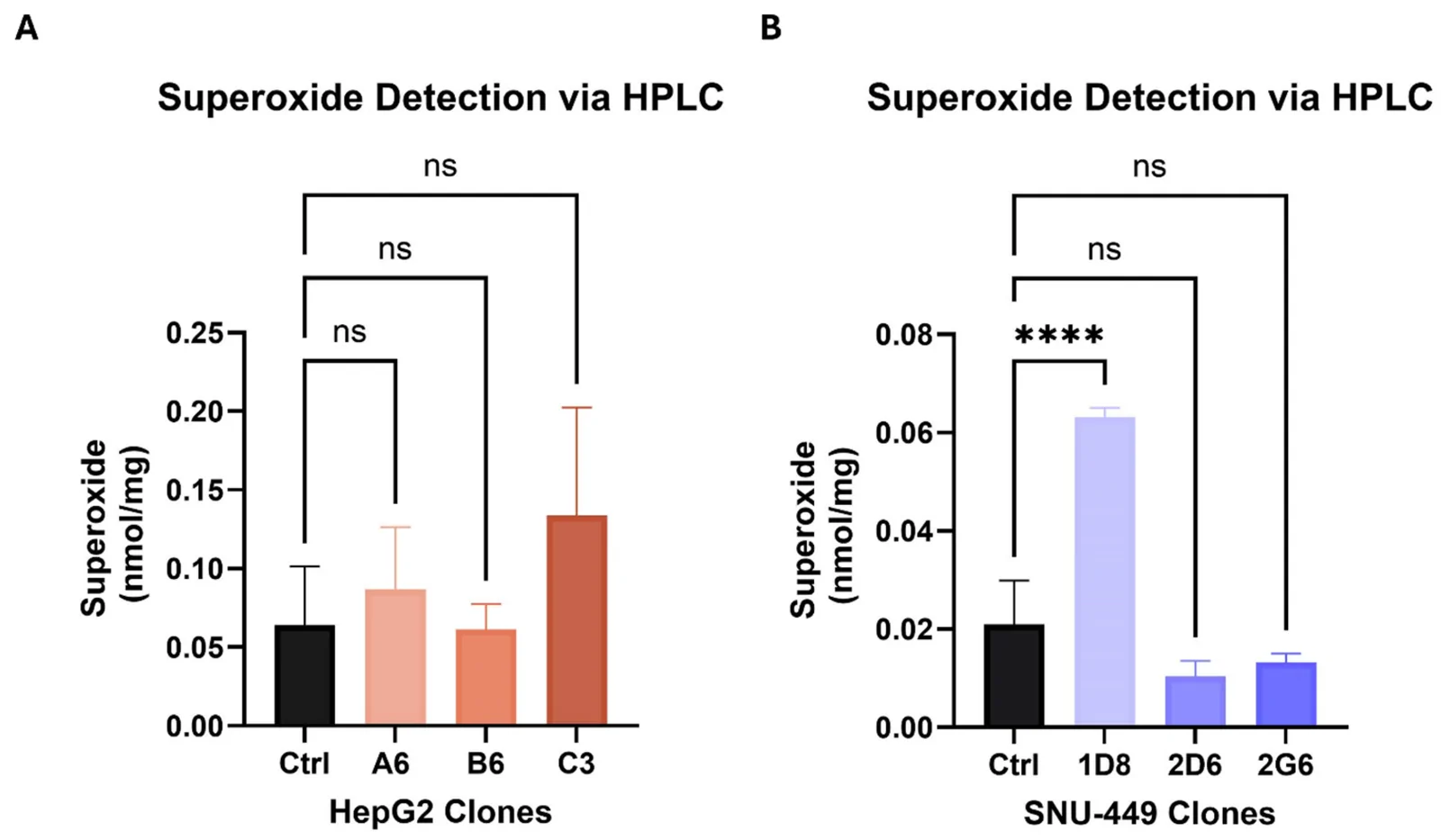
Effects of ADCK1 Knockout on Mitochondrial Superoxide Measured by HPLC. Mitochondrial superoxide production was assessed by high-performance liquid chromatography (HPLC) in HepG2 (**A**) and SNU-449 (**B**) cells. ADCK1 KO did not consistently alter mitochondrial superoxide levels across the majority of KO clones. However, a significant increase was observed in the SNU-449 1D8 clone compared with control cells (**** *p* < 0.0001). ns: no significant difference.

To determine whether these alterations in mitochondrial function were accompanied by changes in mitochondrial reactive oxygen species (ROS) production, we first quantified mitochondrial superoxide by hydroethidine-based HPLC analysis (N = 3). Despite the pronounced defects in mitochondrial respiration, ATP production, and membrane potential observed in ADCK1 knockout cells, mitochondrial superoxide levels remained largely unchanged across most HepG2 and SNU-449 knockout clones (Figure 11A,B). The only notable exception was the SNU-449 1D8 clone, which exhibited a significant increase in mitochondrial superoxide compared with vector control cells (**** *p* < 0.0001).

**Figure 12:**
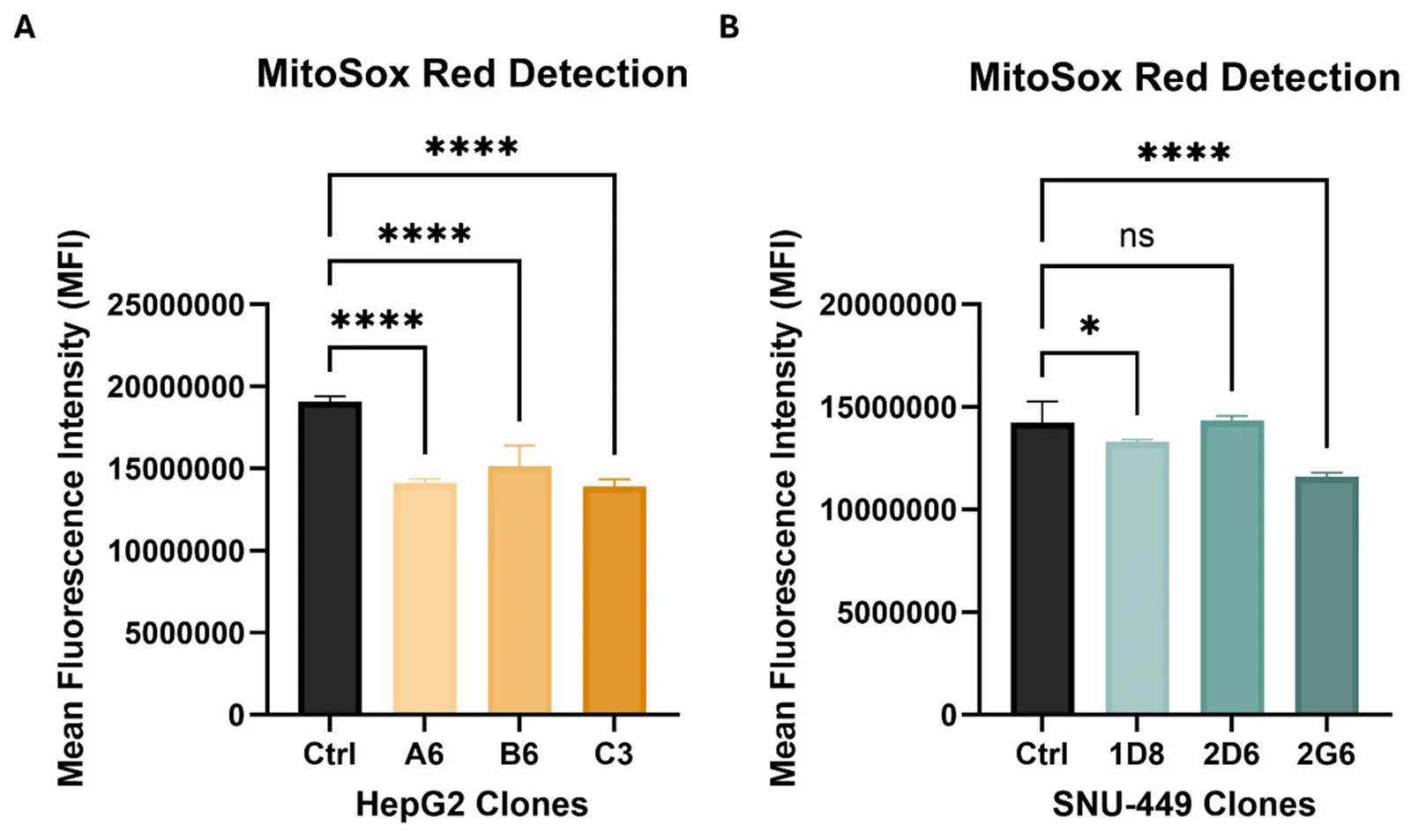
ADCK1 Knockout Reduces Mitochondrial Superoxide Detected by MitoSOX Staining. Mitochondrial superoxide levels were quantified by MitoSOX staining followed by flow cytometric analysis. In HepG2 cells (**A**), all ADCK1 KO clones exhibited significantly reduced MitoSOX fluorescence compared with controls (all **** *p* < 0.0001). In SNU-449 cells (**B**), significant reductions were observed in the 1D8 (* *p* < 0.05) and 2G6 (**** *p* < 0.0001) ADCK1 KO clones. ns: no significant difference.

Because HPLC analysis showed variable responses among individual clones, we further evaluated mitochondrial superoxide production using MitoSOX Red fluorescence and flow cytometry (N = 5–6). In HepG2 cells, ADCK1 knockout produced a consistent and significant reduction in MitoSOX fluorescence across all knockout clones (Figure 12A; all **** *p* < 0.0001), indicating decreased mitochondrial superoxide production. In SNU-449 cells, decreased mitochondrial superoxide levels were observed in the 1D8 (* *p* < 0.05) and 2G6 (**** *p* < 0.0001) clones, whereas the 2D6 clone was not significantly different from the vector control (Figure 12B).

Overall, the HPLC and flow cytometric analyses indicate that ADCK1 knockout does not consistently alter mitochondrial superoxide production in hepatocellular carcinoma cells. Although HPLC analysis identified a significant increase in mitochondrial superoxide in the SNU-449 1D8 clone, MitoSOX Red analysis demonstrated a more consistent reduction in mitochondrial superoxide fluorescence across HepG2 knockout clones and in selected SNU-449 clones. This is because MitoSOX flow cytometry measures total red fluorescence intensity that cannot reliably differentiate superoxide from other oxidants, such as hydrogen peroxide. Despite some variability between assays and among individual clones, the overall findings suggest that ADCK1 KO produces only modest, cell line-dependent changes in mitochondrial superoxide levels.

## 3. Discussion

The present study identifies ADCK1 as a previously unrecognized regulator of mitochondrial bioenergetics in hepatocellular carcinoma (HCC). Using CRISPR/Cas9-mediated gene knockout in two independent HCC cell lines, we have demonstrated that ADCK1 KO produces coordinated defects in oxidative phosphorylation, glycolytic metabolism, ATP production, lactate generation, and mitochondrial membrane potential. Together, these findings establish that ADCK1 is required for maintaining mitochondrial function and cellular energy metabolism in HCC cells. Interestingly, despite these profound metabolic alterations, ADCK1 KO produced only modest and cell line-dependent changes in mitochondrial superoxide production, suggesting that disruption of mitochondrial bioenergetics rather than oxidative stress represents the predominant consequence of ADCK1 KO.

One of the most striking findings in this study was the global reduction in mitochondrial respiration following ADCK1 KO. The consistent decrease in oxygen consumption throughout the mitochondrial stress test indicates that ADCK1 KO broadly impairs oxidative phosphorylation rather than disrupting a single respiratory complex or metabolic pathway. Reductions in basal respiration, ATP-linked respiration, and maximal respiratory capacity collectively suggest that ADCK1 is required to sustain mitochondrial energy production under both physiological and metabolically demanding conditions. Furthermore, the decrease in ATP-linked respiration indicates that diminished oxygen consumption primarily reflects impaired ATP synthesis rather than increased proton leak or non-mitochondrial oxygen consumption. These findings position ADCK1 as an important regulator of mitochondrial respiratory efficiency in HCC cells.

Mitochondrial metabolism has emerged as a critical determinant of hepatocellular carcinoma progression. Although the Warburg hypothesis proposed that cancer cells rely predominantly on aerobic glycolysis for ATP production, it is now well established that many tumors, including HCC, retain substantial dependence on mitochondrial oxidative phosphorylation (OXPHOS) to support proliferation, survival, and metastatic progression [27]. Increased OXPHOS activity has been associated with enhanced tumor aggressiveness, therapeutic resistance, and poor clinical outcomes in HCC, and the expression levels of OXPHOS-related genes have demonstrated prognostic value in patients with liver cancer. The marked suppression of mitochondrial respiration observed following ADCK1 KO therefore suggests that ADCK1 contributes to the metabolic adaptations that support HCC progression [28]. As a result, ADCK1 may represent both a biomarker of mitochondrial metabolic activity and a potential therapeutic target in hepatocellular carcinoma.

In addition to impairing oxidative phosphorylation, ADCK1 KO significantly reduced glycolytic activity, as demonstrated by decreases in the extracellular acidification rates and intracellular lactate levels. Rather than the compensatory increase in glycolysis following mitochondrial dysfunction, ADCK1 knockout cells show simultaneous suppression of both major ATP-producing pathways. This finding is particularly noteworthy because many cancer cells compensate for impaired mitochondrial function by increasing aerobic glycolysis, a phenomenon commonly referred to as the Warburg effect. This metabolic plasticity allows tumor cells to maintain ATP production and generate biosynthetic intermediates necessary for rapid proliferation [29,30,31,32]. In contrast, ADCK1-KO cells were unable to mount this compensatory response and instead adopted a metabolically quiescent phenotype characterized by reduced oxidative phosphorylation, diminished glycolysis, and decreased ATP production. The inability to maintain metabolic flexibility may contribute to the reduced malignant phenotype previously observed following ADCK1 KO and suggests that ADCK1 functions upstream of metabolic pathways required for bioenergetic adaptation.

The reductions in intracellular ATP provide additional biochemical evidence supporting this interpretation. Independent ATP measurements closely paralleled the decreases observed in basal and ATP-linked respiration, confirming that impaired mitochondrial function translates directly into reduced cellular energy production. These findings further strengthen the conclusion that ADCK1 is essential for maintaining bioenergetic homeostasis in HCC cells. Interestingly, similar metabolic mechanisms have recently been described for the mitochondrial RNA polymerase POLRMT, another mitochondrial regulator associated with poor prognosis in HCC. POLRMT promotes oxidative phosphorylation and ATP production by activating the AMPK–caspase-3 signaling pathway, and pharmacological inhibition of POLRMT by IMT1B restores lenvatinib sensitivity in both in vitro and in vivo HCC models. These observations raise the intriguing possibility that ADCK1 may function within related mitochondrial regulatory pathways or influence therapeutic sensitivity through similar bioenergetic mechanisms. Future studies examining interactions between ADCK1 and established regulators of mitochondrial metabolism, including AMPK, PGC-1α, TFAM, and POLRMT, may provide important mechanistic insight [33].

Maintenance of mitochondrial membrane potential (Δψm) is critical for oxidative phosphorylation, ATP production, mitochondrial protein import, and overall organelle homeostasis. The electron transport chain establishes this electrochemical gradient across the inner mitochondrial membrane, which drives ATP synthesis. Disruption of Δψm is therefore a key indicator of mitochondrial dysfunction and is frequently associated with impaired bioenergetics and metabolic collapse [5,11]. ADCK1 knockout caused a decrease in TMRE fluorescence intensity in the HepG2 A6 and B6 clones and in the SNU-449 2D6 relative to vector control cells, consistent with partial depolarization of the mitochondrial membrane in these subpopulations. However, this effect was not uniformly observed across all ADCK1 knockout clones, indicating variability in the extent of mitochondrial membrane perturbation following ADCK1 loss. This clone-dependent response suggests that ADCK1 KO does not universally compromise Δψm under the tested conditions, which may sensitize specific cellular contexts or genetic backgrounds to alterations in mitochondrial polarization.

Despite this variability, the observed reductions in TMRE fluorescence in select clones are consistent with impaired mitochondrial function and align with the decreases in oxidative phosphorylation and ATP production identified in this study. Together, these findings suggest that ADCK1 contributes, at least in part, to the maintenance of mitochondrial membrane polarization and bioenergetic stability in hepatocellular carcinoma cells. However, its effects on Δψm appear to be context-dependent rather than uniformly penetrant across all knockout clones.

Surprisingly, mitochondrial superoxide production was not consistently increased following ADCK1 KO, despite significant impairments in mitochondrial respiration and membrane potential. Our HPLC analysis did not reveal consistent changes in mitochondrial superoxide production across the majority of ADCK1 knockout clones. Several factors may explain the limited differences observed with this method. Firstly, the relatively small sample size (N = 3) could have influenced the results. Next, HPLC quantifies superoxide-specific oxidation products from whole-cell lysates, providing an average measurement across the entire cell population, which may obscure variations between individual cells. In contrast, flow cytometry allows for the analysis of individual cells and may therefore be more sensitive to subtle changes within heterogeneous cell populations.

Our MitoSOX Red flow cytometric analysis demonstrated a more reproducible reduction in mitochondrial superoxide levels following ADCK1 KO. In HepG2 cells, all knockout clones exhibited significantly lower MitoSOX fluorescence than vector controls, whereas in SNU-449 cells significant reductions were observed in the 1D8 and 2G6 clones, with the 2D6 clone remaining unchanged. The greater reduction observed in HepG2 cells may reflect intrinsic differences in mitochondrial metabolism or redox homeostasis between the two HCC cell lines. It should also be noted that MitoSOX accumulation is positively correlated with mitochondrial membrane potential, which was significantly reduced in ADCK1 knockout cells. When interpreted alongside the Seahorse, ATP, and TMRE data, these findings suggest that ADCK1 KO primarily impairs mitochondrial bioenergetics rather than promoting excessive mitochondrial oxidative stress. Reduced electron transport chain activity after ADCK1 KO may also limit electron leakage from respiratory complexes I and III, thereby decreasing mitochondrial superoxide generation despite mitochondrial dysfunction [11,34,35].

These findings warrant further investigation. Current systemic therapies for advanced HCC, including multikinase inhibitors such as lenvatinib and sorafenib, provide only modest improvements in overall survival, and resistance remains a major clinical challenge. Consequently, there is growing interest in identifying metabolic vulnerabilities that can be exploited therapeutically [3,6]. Our findings suggest that ADCK1 represents one such vulnerability by simultaneously suppressing oxidative phosphorylation and glycolysis, thereby limiting the metabolic flexibility that is essential for tumor survival. Although selective ADCK1 inhibitors have not yet been developed, small-molecule inhibitors targeting the closely related family member ADCK3 have demonstrated that ATPase atypical kinase (ADCK) proteins are pharmacologically tractable. Given the high degree of structural conservation within the ADCK protein family, these studies provide a rationale for structure-guided development of selective ADCK1 inhibitors. Such compounds may ultimately prove useful either as single-agent therapies or in combination with existing HCC treatments to overcome metabolic adaptation and drug resistance [20,36,37].

Several limitations of the present study should be acknowledged. First, although we establish a clear role for ADCK1 in maintaining mitochondrial bioenergetics, the molecular mechanisms underlying these effects remain undefined. Future studies should determine whether ADCK1 directly regulates mitochondrial respiratory complexes, coenzyme Q biosynthesis, mitochondrial dynamics, or signaling pathways involved in metabolic homeostasis. In the future, we will determine whether and how ADCK1 regulates coenzyme Q biosynthesis, mitochondrial fusion/fission, and key regulatory proteins to elucidate underlying mechanisms. Second, rescue experiments to confirm on-target effects were not conducted. Third, all experiments were performed using in vitro cell culture models. Validation in xenograft or other types of mouse models will be necessary to determine whether ADCK1 similarly regulates tumor growth and metabolism in vivo. Finally, while our data demonstrate significant metabolic alterations following ADCK1 KO, additional studies evaluating mitochondrial fusion/fission, mitochondrial DNA copy number, and mitochondrial ultrastructure would further clarify ADCK1’s contribution to mitochondrial homeostasis.

## 4. Materials and Methods

### 4.1. Cell Culture and Reagents

Human hepatocellular carcinoma cell lines HepG2 and SNU-449 (The American Type Culture Collection, Manassas, VA, USA, HB-8065 and CRL-2234, respectively) were cultured and transfected using a CRISPR-lentivirus system (Applied Biological Materials (ABM), Richmond, BC, Canada, cat log #: 113851110502) under standard conditions. HepG2 cells were maintained in Eagle’s Minimum Essential Medium (EMEM; Thermo Fisher Scientific, Waltham, MA, USA) supplemented with 10% fetal bovine serum (FBS), 1% L-glutamine, 0.4% penicillin, and 0.2% Plasmocin. SNU-449 cells were cultured in Roswell Park Memorial Institute (Buffalo, NY, USA) (RPMI-1640) medium supplemented with identical concentrations of FBS, L-glutamine, penicillin, and Plasmocin. All cells were incubated at 37 °C in a humidified atmosphere containing 5% CO_2_. ADCK1 KO cells were characterized in a previous study.

### 4.2. Measurement of Oxygen Consumption Rate (OCR) and Extracellular Acidification Rate (ECAR)

An Agilent Seahorse XF24 Extracellular Flux Analyzer (Santa Clara, CA, USA) was used to measure mitochondrial respiration and glycolytic activity. Cells were seeded into Seahorse XF24 V7 PS Cell Culture Microplates (part number 100777-004) at a density of 1 × 10^4^ cells per well and allowed to adhere overnight (16–18 h). One hour to analysis, culture medium was replaced with serum- and dye-free Agilent Seahorse DMEM (HepG2; part number 103575-100) or RPMI (SNU-449; part number 103576-100) assay medium. Experiments were performed using a Seahorse XF Cell Mito Stress Test kit (part number 103015-100) (Agilent) and a Seahorse XFe24 FluxPak (part number 102340-100). During the mitochondrial stress test, oligomycin (5 μM), FCCP (1.25 μM), and rotenone/antimycin A (5 μM) were sequentially injected to inhibit ATP synthase, uncouple oxidative phosphorylation, and inhibit mitochondrial electron transport complexes I and III, respectively. OCR and ECAR were calculated using Agilent Wave Desktop software (2.4) and normalized to total cellular protein.

### 4.3. Intracellular ATP Quantification

The Cayman ATP Detection Assay Kit (Item No. 700410) (Ann Arbor, MI, USA) was used to detect the cellular ATP levels. Cells were seeded into 6-well plates (3 × 10^5^ cells per well) and harvested at approximately 50% confluence. Following washing with ice-cold PBS, cells were lysed in ATP Detection Sample Buffer (Item No. 700411) and maintained on ice throughout sample preparation. ATP standards were prepared according to the manufacturer’s protocol. Samples (10 μL) and ATP standards were dispensed into white 96-well plates, followed by 100 μL luciferase reaction mixture containing luciferase, D-luciferin, and DTT reagent. Luminescence was measured after incubation for 15 min at room temperature in the dark using a BioTek Synergy H1 microplate reader. ATP concentrations were calculated from a standard curve generated using the accompanying software.

### 4.4. Cellular Lactate Assay

The Abcam L-Lactate Colorimetric Assay Kit (ab65331) (Novi, MI, USA) was used to measure the intracellular lactate levels. Cells were cultured overnight in T75 flasks, harvested, and resuspended at 2 × 10^6^ cells/mL in ice-cold PBS. Cell pellets were lysed in assay buffer, homogenized by pipetting, and clarified by centrifugation (10,000× *g*, 5 min, 4 °C). Samples and lactate standards (50 μL) were transferred to a 96-well plate, followed by adding the reaction mixture. After incubation for 30 min at room temperature in the dark, absorbance was measured at 450 nm using a BioTek Synergy H1 microplate reader. Lactate concentrations were calculated from the standard curve according to the manufacturer’s recommended equation and corrected for sample dilution. Sample calculations were generated using the manufacturer’s recommended equation:

*Lactate concentration* = (*La*/*Sv*) × *D*

where La = amount of Lactic acid in the sample well calculated from the standard curve (nmol). Sv = volume of sample added into the well (μL). D = sample dilution factor.

### 4.5. Mitochondrial Membrane Potential Assay

Mitochondrial membrane potential was determined using the G-Biosciences (St. Louis, MO, USA) TMRE Mitochondrial Membrane Potential Assay Kit (Cat. Nos. 786-1313 and 786-1314). Cells were seeded into black, clear-bottom 96-well plates at 1 × 10^4^ cells per well and cultured overnight. Cells were incubated with 500 nM tetramethylrhodamine ethyl ester (TMRE) for 30 min at 37 °C in the dark, washed with PBS containing 0.2% bovine serum albumin (BSA), and fluorescence was measured using a BioTek Synergy H1 microplate reader (Shoreline, WA, USA) (Ex/Em = 549/575 nm). The relative TMRE Fluorescence (%) was calculated according to the manufacturer’s recommended equation:

Relative TMRE Fluorescence (%) = (Sample RFU − Blank RFU) × 100/(Baseline RFU − Blank RFU)

where Sample RFU= Fluorescence of cells treated with drug or chemical compound; Blank RFU = Fluorescence of microwells with no cells baseline vector control); and Baseline Vector Control RFU = Fluorescence of cells not treated with drug/chemical compound. The relative TMRE fluorescence levels in fold change were subsequently calculated.

### 4.6. Mitochondrial Superoxide Detection via HPLC

Mitochondrial superoxide production was quantified by the LSU Health Shreveport Analytical Redox Biology Sub-Core using hydroethidine (HE)-based HPLC analysis. Cells were incubated with 10 μM HE for 30 min, washed twice with ice-cold Dulbecco’s phosphate-buffered saline (DPBS), harvested by scraping using ice-cold DPBS, and pelleted by centrifugation (1000× *g*, 5 min, 4 °C). Cell pellets were stored at −80 °C until analysis. At the core facility, pellets were lysed in DPBS containing 0.1% Triton X-100 and clarified by centrifugation (12,000× *g*, 8 min, 4 °C). Proteins were precipitated using methanol/perchloric acid, and the pH was adjusted with 1 M phosphate buffer (pH 2.6) to precipitate potassium perchlorate. After centrifugation, clarified supernatants were stored at −20 °C before HPLC analysis. Protein concentrations were calculated before sample precipitation for normalization.

### 4.7. Mitochondrial Superoxide Detection via Flow Cytometry

Mitochondrial superoxide production was also assessed using MitoSOX™ Red (Invitrogen (Carlsbad, CA, USA); Cat. No. M36008). Cells were incubated with 500 nM MitoSOX Red for 30 min at 37 °C, protected from light. Following incubation, cells were washed three times with warm DPBS, harvested, resuspended in DPBS, and analyzed by flow cytometry. Cells treated with 30% hydrogen peroxide served as positive controls (final concentration 100 µM). Fluorescence intensity was measured using identical instrument settings for all experimental groups.

### 4.8. Statistical Analysis

Statistical analysis was performed using GraphPad Prism v11. All experiments were repeated at least three times. Data are presented as mean ± SD. We performed one-way ANOVA for multiple group comparisons, followed by Dunnett’s multiple comparisons test with a 95% confidence interval.

## 5. Conclusions

In conclusion, this study identifies ADCK1 as an important regulator of mitochondrial bioenergetics in hepatocellular carcinoma. ADCK1 KO resulted in coordinated impairment of oxidative phosphorylation, glycolysis, ATP production, lactate generation, and mitochondrial membrane potential across HCC cell models. Notably, these metabolic defects occurred without a consistent increase in mitochondrial superoxide production, suggesting that ADCK1 primarily supports mitochondrial energy metabolism and bioenergetic homeostasis rather than functioning predominantly through regulating oxidative stress.

The simultaneous suppression of oxidative phosphorylation and glycolysis following ADCK1 loss further indicates that ADCK1 may be required for the metabolic functions that enable HCC cells to adapt to energetic stress. Disruption of this metabolic capacity may contribute to the reduced tumorigenic phenotype associated with ADCK1 deficiency and highlights a potential metabolic vulnerability that could be therapeutically exploited. Although the molecular mechanisms linking ADCK1 to mitochondrial function remain to be established, these findings provide a foundation for investigating its interactions with established regulators of mitochondrial metabolism and respiratory function.

Collectively, our findings establish ADCK1 as a previously unrecognized component of HCC metabolic regulation and support its potential utility as both a biomarker of mitochondrial bioenergetic activity and a therapeutic target. Future studies using mechanistic, pharmacological, and in vivo approaches will be essential to determine how ADCK1 regulates mitochondrial function and whether targeting ADCK1 can impair tumor growth or enhance the efficacy of existing HCC therapies.

## Data Availability

All data generated or analyzed during this study are included in this published article.
